# Supplementary figures and images for: NF-YA transcriptionally activates the expression of SOX2 in cervical cancer stem cells
Source: PLoS One. 2019 Jul 31;14(7):e0215494. doi: 10.1371/journal.pone.0215494 (PMC6668781; doi:10.1371/journal.pone.0215494)

EGFP-  
NF-YA

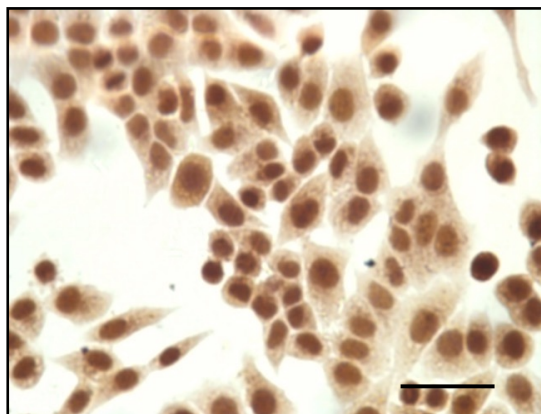

C33A

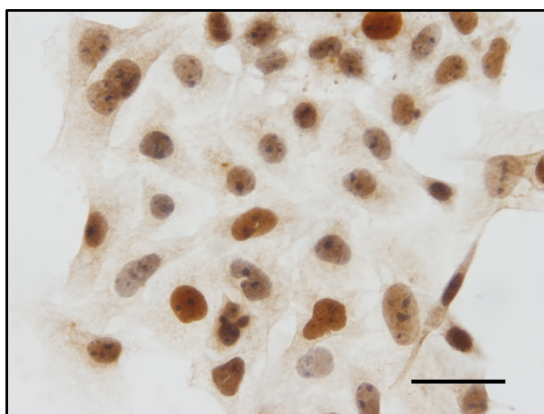

EGFP-  
Control

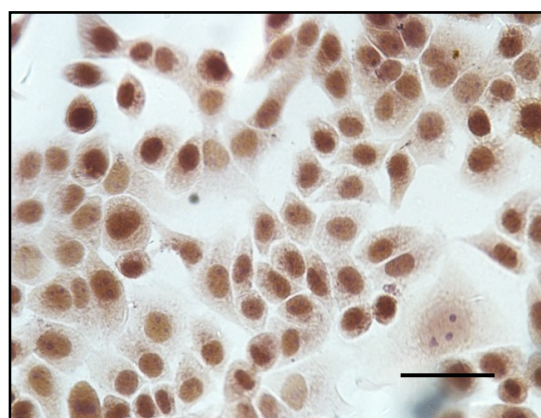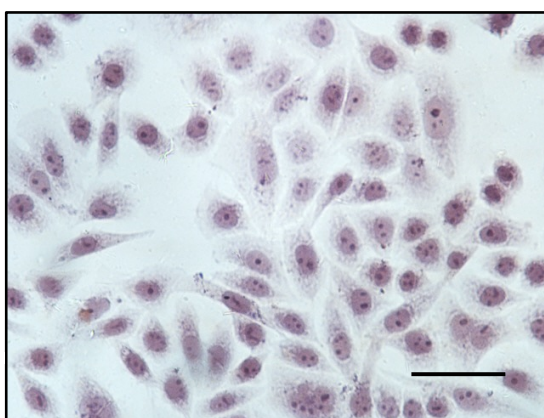

Supplement: S1 Fig — The expression of OCT4 protein was detected by IHC in EGFP-NF-YA and GEFP-Control groups of SiHa and C33A cells. (PDF) [file pone.0215494.s001.pdf]
